# 2-[2-(Hydroxy­meth­yl)phen­yl]-1-phenyl­ethanol

**DOI:** 10.1107/S1600536809003043

**Published:** 2009-01-28

**Authors:** P. Manivel, Venkatesha R. Hathwar, S. Mohanaroopan, K. Prabakaran, F. Nawaz Khan

**Affiliations:** a Chemistry Division, School of Science and Humanities, VIT University, Vellore 632 014, Tamil Nadu, India; b Solid State and Structural Chemistry Unit, Indian Institute of Science, Bangalore 560 012, Karnataka, India

## Abstract

The title compound, C_15_H_16_O_2_, has a dihedral angle of 19.10 (5)° between the mean planes of the two benzene rings. There is an intra­molecular O—H⋯O hydrogen bond and the C—C—C—C torsion angle across the bridge between the two rings is 173.13 (14)°. The mol­ecules form inter­molecular O—H⋯O hydrogen-bonded chains extending along the *a* axis. C—H⋯π contacts are also observed between mol­ecules within the chains.

## Related literature

For bond lengths in organic compounds, see: Allen *et al.* (1987 ▶). For general background, see: Azzena *et al.* (1996 ▶), and references therein; Barluenga *et al.* (1987 ▶); Shing *et al.* (1994 ▶); Lim & Hudson (2004 ▶); Tirodkar & Usgaonkar (1972 ▶); Odabaşoglu *et al.* (2007 ▶). For related crystal structures, see: Gałdecki *et al.* (1984 ▶); Hoyos-Guerrero *et al.* (1983 ▶).
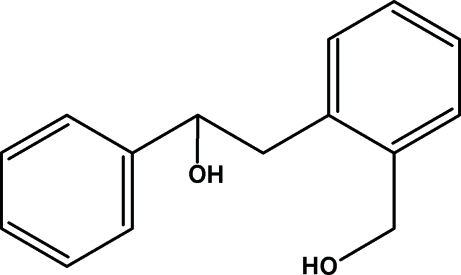


## Experimental

### 

#### Crystal data

C_15_H_16_O_2_
*M* *_r_* = 228.28
Orthorhombic, 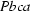

*a* = 8.550 (1) Å
*b* = 15.8676 (18) Å
*c* = 18.593 (2) Å
*V* = 2522.4 (5) Å^3^
*Z* = 8
Mo *K*α radiation
μ = 0.08 mm^−1^
*T* = 290 (2) K
0.33 × 0.30 × 0.05 mm

#### Data collection

Bruker SMART CCD area-detector diffractometer
Absorption correction: multi-scan (*SADABS*; Sheldrick, 1996 ▶) *T*_min_ = 0.941, *T*_max_ = 0.996
17664 measured reflections
2347 independent reflections
1618 reflections with *I* > 2σ(*I*)
*R*_int_ = 0.051

#### Refinement

*R*[*F* ^2^ > 2σ(*F* ^2^)] = 0.048
*wR*(*F* ^2^) = 0.100
*S* = 1.05
2347 reflections
218 parameters
All H-atom parameters refined
Δρ_max_ = 0.17 e Å^−3^
Δρ_min_ = −0.26 e Å^−3^

### 

Data collection: *SMART* (Bruker, 2004 ▶); cell refinement: *SAINT* (Bruker, 2004 ▶); data reduction: *SAINT*; program(s) used to solve structure: *SHELXS97* (Sheldrick, 2008 ▶); program(s) used to refine structure: *SHELXL97* (Sheldrick, 2008 ▶); molecular graphics: *ORTEP-3* (Farrugia, 1997 ▶) and *CAMERON* (Watkin *et al.*, 1993 ▶); software used to prepare material for publication: *PLATON* (Spek, 2003 ▶).

## Supplementary Material

Crystal structure: contains datablocks global, I. DOI: 10.1107/S1600536809003043/si2148sup1.cif

Structure factors: contains datablocks I. DOI: 10.1107/S1600536809003043/si2148Isup2.hkl

Additional supplementary materials:  crystallographic information; 3D view; checkCIF report

## Figures and Tables

**Table 1:** Hydrogen-bond geometry (Å, °) *Cg*2 is the centroid of the C9–C14 ring.

*D*—H⋯*A*	*D*—H	H⋯*A*	*D*⋯*A*	*D*—H⋯*A*
O2—H2O⋯O1^i^	0.86 (2)	1.89 (2)	2.745 (2)	170.5 (24)
O1—H1O⋯O2	0.93 (2)	1.78 (2)	2.706 (2)	173.6 (22)
C15—H15*B*⋯*Cg*2^i^	0.95 (2)	2.638 (18)	3.504 (2)	151.7 (14)

Symmetry code: (i) 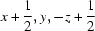.

**Fig. 1.**
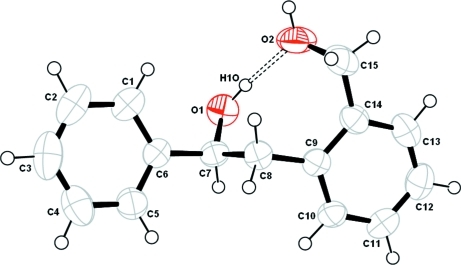
ORTEP diagram of molecule (I) with 50% probability displacement ellipsoids. The dotted lines indicates O—H···O intramolecular hydrogen bond.

**Fig. 2.**
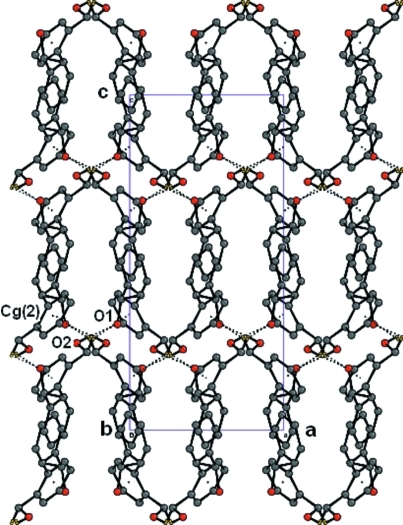
The crystal packing diagram of (I).The dotted lines indicate intermolecular hydrogen bonds. All H atoms have been omitted for clarity.

## supplementary crystallographic information

### Comment

A wide range of diaryl diols have been prepared earlier from phthalane and readily available substituted benzaldehyde (Azzena *et al.*, 1996). The diols in general can act as precursors of corresponding oxygen containing heterocyclic compounds by a dehydration process for *e.g.* benzodihydropyrans; benzoxepines have been prepared (Barluenga *et al.*, 1987, Shing *et al.*, 1987). The hydroxyl structural moiety was found in numerous pharmaceutically active compounds and therefore represents an interesting template for combinatorial as well as medicinal chemistry (Lim and Hudson, 2004). In particular phenylethanol derivatives have good antifungal properties (Tirodkar and Usgaonkar, 1972, Odabaşoglu *et al.*, 2007, Gałdecki *et al.*, 1984, Hoyos-Guerrero *et al.*, 1983).

All the bond lengths are within normal ranges in the title compound (Fig. 1) (Allen *et al.*, 1987). The tight conformation of the molecule is held by an O—H···O intramolecular hydrogen bond (Fig. 1) with C6—C7—C8—C9 torsional angle of 173.13 (14)°. Further, O—H···O and C—H···π (Fig. 2) intermolecular interactions stabilize the packing of the crystal structure and form chains running along the *a* axis. Cg2 is the centroid of the hydroxymethylphenyl ring C9 - C14 (Table 1).

### Experimental

3-Phenylisocoumarin (1 eq.) was dissolved in 10 volumes of methanol, sodium borohydride (4 eq.) was added to it and stirred at 50° C under nitrogen atmosphere for 4 hrs. Then two more equivalents of NaBH_4_ was further added and left overnight at 50° C for completion of the reaction. After TLC analysis, solvent methanol was removed, extracted with ethyl acetate. The ethyl acetate layer was washed with water, dried with anhydrous Na_2_SO_4_, evaporated to yield the title compound, which was further purified by washing with petroleum ether. Single-crystals for the structure analysis were obtained by slow evaporation of the ethanol solution.

### Refinement

All H atoms of (I) were located from a difference Fourier map and refined isotropically [C—H = 0.937 (18) - 1.005 (16) Å and O—H = 0.87 (2) - 0.93 (2) Å] and *U*_iso_(H) = 1.2*U*_eq_(C) for all H atoms.

### Crystal data

**Table:**

C_15_H_16_O_2_	*F*(000) = 976
*M**_r_* = 228.28	*D*_x_ = 1.202 Mg m^−^^3^
Orthorhombic, *P**b**c**a*	Mo *K*α radiation, λ = 0.71073 Å
Hall symbol: -P 2ac 2ab	Cell parameters from 2451 reflections
*a* = 8.550 (1) Å	θ = 2.6–19.6°
*b* = 15.8676 (18) Å	µ = 0.08 mm^−^^1^
*c* = 18.593 (2) Å	*T* = 290 K
*V* = 2522.4 (5) Å^3^	Plate, colorless
*Z* = 8	0.33 × 0.30 × 0.05 mm

### Data collection

**Table:**

Bruker SMART CCD area-detector diffractometer	2347 independent reflections
Radiation source: fine-focus sealed tube	1618 reflections with *I* > 2σ(*I*)
graphite	*R*_int_ = 0.051
φ and ω scans	θ_max_ = 25.5°, θ_min_ = 2.2°
Absorption correction: multi-scan (*SADABS*; Sheldrick, 1996)	*h* = −10→10
*T*_min_ = 0.941, *T*_max_ = 0.996	*k* = −19→19
17664 measured reflections	*l* = −21→22

### Refinement

**Table:**

Refinement on *F*^2^	Primary atom site location: structure-invariant direct methods
Least-squares matrix: full	Secondary atom site location: difference Fourier map
*R*[*F*^2^ > 2σ(*F*^2^)] = 0.048	Hydrogen site location: inferred from neighbouring sites
*wR*(*F*^2^) = 0.100	All H-atom parameters refined
*S* = 1.05	*w* = 1/[σ^2^(*F*_o_^2^) + (0.042*P*)^2^ + 0.2211*P*] where *P* = (*F*_o_^2^ + 2*F*_c_^2^)/3
2347 reflections	(Δ/σ)_max_ < 0.001
218 parameters	Δρ_max_ = 0.17 e Å^−^^3^
0 restraints	Δρ_min_ = −0.26 e Å^−^^3^

### Special details

**Table:**

Geometry. All esds (except the esd in the dihedral angle between two l.s. planes) are estimated using the full covariance matrix. The cell esds are taken into account individually in the estimation of esds in distances, angles and torsion angles; correlations between esds in cell parameters are only used when they are defined by crystal symmetry. An approximate (isotropic) treatment of cell esds is used for estimating esds involving l.s. planes.
Refinement. Refinement of *F*^2^ against ALL reflections. The weighted *R*-factor *wR* and goodness of fit *S* are based on *F*^2^, conventional *R*-factors *R* are based on *F*, with *F* set to zero for negative *F*^2^. The threshold expression of *F*^2^ > σ(*F*^2^) is used only for calculating *R*-factors(gt) *etc*. and is not relevant to the choice of reflections for refinement. *R*-factors based on *F*^2^ are statistically about twice as large as those based on *F*, and *R*- factors based on ALL data will be even larger.

### Fractional atomic coordinates and isotropic or equivalent isotropic displacement parameters (Å^2^)

**Table:**

	*x*	*y*	*z*	*U*_iso_*/*U*_eq_	
O1	−0.08023 (15)	0.19969 (8)	0.31581 (6)	0.0587 (4)	
O2	0.15538 (17)	0.12736 (10)	0.24081 (8)	0.0790 (5)	
C1	0.0686 (2)	0.32636 (11)	0.40207 (12)	0.0631 (5)	
C2	0.1106 (3)	0.39491 (13)	0.44389 (16)	0.0791 (7)	
C3	0.0712 (3)	0.39837 (14)	0.51474 (16)	0.0791 (7)	
C4	−0.0117 (2)	0.33381 (15)	0.54520 (14)	0.0743 (6)	
C5	−0.0560 (2)	0.26587 (13)	0.50386 (11)	0.0616 (5)	
C6	−0.01577 (17)	0.26096 (10)	0.43191 (9)	0.0461 (4)	
C7	−0.05662 (19)	0.18243 (10)	0.39035 (9)	0.0469 (4)	
C8	0.0685 (2)	0.11513 (10)	0.40226 (10)	0.0478 (4)	
C9	0.03113 (17)	0.03032 (10)	0.37050 (8)	0.0445 (4)	
C10	−0.0763 (2)	−0.02101 (11)	0.40534 (9)	0.0514 (4)	
C11	−0.1144 (2)	−0.09990 (12)	0.38015 (11)	0.0626 (5)	
C12	−0.0437 (3)	−0.12940 (13)	0.31873 (12)	0.0692 (6)	
C13	0.0619 (2)	−0.07976 (13)	0.28324 (11)	0.0647 (5)	
C14	0.10119 (19)	0.00024 (11)	0.30772 (8)	0.0518 (4)	
C15	0.2216 (2)	0.05066 (14)	0.26765 (12)	0.0692 (6)	
H1O	0.004 (3)	0.1787 (13)	0.2901 (11)	0.096 (8)*	
H2O	0.232 (3)	0.1551 (14)	0.2219 (11)	0.105 (8)*	
H1	0.101 (2)	0.3201 (12)	0.3512 (10)	0.079 (6)*	
H2	0.171 (3)	0.4390 (14)	0.4206 (11)	0.104 (7)*	
H3	0.100 (2)	0.4462 (13)	0.5457 (11)	0.093 (7)*	
H4	−0.038 (2)	0.3338 (12)	0.5953 (11)	0.087 (7)*	
H5	−0.111 (2)	0.2208 (11)	0.5256 (9)	0.065 (5)*	
H7	−0.1592 (18)	0.1606 (9)	0.4084 (7)	0.044 (4)*	
H8A	0.0803 (17)	0.1090 (9)	0.4542 (9)	0.059 (5)*	
H8B	0.1713 (19)	0.1375 (9)	0.3842 (8)	0.052 (4)*	
H10	−0.1226 (17)	0.0018 (10)	0.4494 (8)	0.053 (4)*	
H11	−0.187 (2)	−0.1344 (11)	0.4052 (9)	0.071 (6)*	
H12	−0.067 (2)	−0.1845 (13)	0.3011 (10)	0.082 (6)*	
H13	0.111 (2)	−0.0980 (11)	0.2409 (10)	0.068 (5)*	
H15A	0.314 (2)	0.0640 (11)	0.3010 (9)	0.076 (6)*	
H15B	0.261 (2)	0.0187 (12)	0.2284 (10)	0.080 (6)*	

### Atomic displacement parameters (Å^2^)

**Table:**

	*U*^11^	*U*^22^	*U*^33^	*U*^12^	*U*^13^	*U*^23^
O1	0.0585 (8)	0.0659 (8)	0.0516 (7)	0.0040 (6)	−0.0152 (6)	0.0044 (6)
O2	0.0660 (9)	0.0919 (11)	0.0791 (10)	0.0021 (8)	0.0273 (8)	0.0233 (8)
C1	0.0620 (12)	0.0552 (12)	0.0722 (14)	−0.0072 (10)	−0.0079 (10)	0.0099 (10)
C2	0.0730 (15)	0.0498 (13)	0.114 (2)	−0.0109 (11)	−0.0164 (14)	0.0105 (14)
C3	0.0643 (14)	0.0566 (13)	0.116 (2)	0.0113 (11)	−0.0255 (14)	−0.0242 (14)
C4	0.0616 (13)	0.0821 (16)	0.0791 (16)	0.0104 (12)	0.0005 (11)	−0.0248 (13)
C5	0.0565 (12)	0.0610 (12)	0.0672 (13)	−0.0047 (10)	0.0067 (10)	−0.0056 (10)
C6	0.0373 (9)	0.0451 (9)	0.0560 (11)	0.0034 (7)	−0.0046 (7)	0.0040 (8)
C7	0.0417 (9)	0.0504 (10)	0.0487 (10)	−0.0038 (8)	−0.0013 (8)	0.0044 (8)
C8	0.0480 (10)	0.0513 (10)	0.0443 (10)	0.0026 (8)	−0.0058 (8)	0.0012 (8)
C9	0.0431 (9)	0.0486 (9)	0.0417 (9)	0.0053 (7)	−0.0065 (7)	0.0050 (7)
C10	0.0589 (11)	0.0525 (11)	0.0429 (10)	0.0048 (9)	0.0004 (8)	0.0092 (8)
C11	0.0669 (13)	0.0549 (12)	0.0661 (13)	−0.0063 (10)	−0.0037 (10)	0.0141 (10)
C12	0.0813 (15)	0.0502 (12)	0.0760 (14)	−0.0033 (11)	−0.0132 (12)	−0.0065 (11)
C13	0.0686 (13)	0.0682 (13)	0.0573 (12)	0.0092 (11)	0.0011 (10)	−0.0147 (10)
C14	0.0462 (10)	0.0587 (11)	0.0505 (10)	0.0059 (8)	0.0004 (8)	−0.0019 (9)
C15	0.0548 (12)	0.0803 (15)	0.0724 (14)	0.0045 (11)	0.0189 (11)	−0.0051 (12)

### Geometric parameters (Å, °)

**Table:**

O1—C7	1.4270 (19)	C7—H7	1.001 (14)
O1—H1O	0.93 (2)	C8—C9	1.504 (2)
O2—C15	1.432 (2)	C8—H8A	0.977 (17)
O2—H2O	0.87 (2)	C8—H8B	1.005 (16)
C1—C6	1.380 (2)	C9—C10	1.388 (2)
C1—C2	1.384 (3)	C9—C14	1.396 (2)
C1—H1	0.990 (18)	C10—C11	1.376 (2)
C2—C3	1.361 (3)	C10—H10	0.979 (15)
C2—H2	0.97 (2)	C11—C12	1.374 (3)
C3—C4	1.369 (3)	C11—H11	0.947 (18)
C3—H3	0.99 (2)	C12—C13	1.368 (3)
C4—C5	1.377 (3)	C12—H12	0.955 (19)
C4—H4	0.96 (2)	C13—C14	1.390 (3)
C5—C6	1.383 (3)	C13—H13	0.937 (18)
C5—H5	0.948 (17)	C14—C15	1.501 (2)
C6—C7	1.507 (2)	C15—H15A	1.024 (19)
C7—C8	1.528 (2)	C15—H15B	0.952 (19)
			
C7—O1—H1O	108.8 (13)	C7—C8—H8A	106.6 (9)
C15—O2—H2O	105.9 (15)	C9—C8—H8B	111.7 (9)
C6—C1—C2	120.0 (2)	C7—C8—H8B	108.5 (9)
C6—C1—H1	117.0 (11)	H8A—C8—H8B	106.0 (12)
C2—C1—H1	122.9 (11)	C10—C9—C14	118.26 (15)
C3—C2—C1	120.8 (2)	C10—C9—C8	118.85 (15)
C3—C2—H2	122.2 (13)	C14—C9—C8	122.88 (15)
C1—C2—H2	117.0 (13)	C11—C10—C9	122.12 (18)
C2—C3—C4	119.9 (2)	C11—C10—H10	121.7 (9)
C2—C3—H3	122.3 (12)	C9—C10—H10	116.2 (9)
C4—C3—H3	117.8 (12)	C12—C11—C10	119.3 (2)
C3—C4—C5	119.8 (2)	C12—C11—H11	119.9 (10)
C3—C4—H4	121.5 (12)	C10—C11—H11	120.8 (10)
C5—C4—H4	118.7 (12)	C13—C12—C11	119.7 (2)
C4—C5—C6	121.0 (2)	C13—C12—H12	120.0 (12)
C4—C5—H5	119.3 (11)	C11—C12—H12	120.3 (12)
C6—C5—H5	119.6 (10)	C12—C13—C14	121.84 (19)
C1—C6—C5	118.44 (17)	C12—C13—H13	121.5 (11)
C1—C6—C7	122.45 (16)	C14—C13—H13	116.7 (11)
C5—C6—C7	118.99 (15)	C13—C14—C9	118.84 (17)
O1—C7—C6	111.84 (13)	C13—C14—C15	119.33 (17)
O1—C7—C8	111.96 (14)	C9—C14—C15	121.79 (17)
C6—C7—C8	109.95 (13)	O2—C15—C14	110.79 (16)
O1—C7—H7	105.5 (8)	O2—C15—H15A	109.8 (11)
C6—C7—H7	108.5 (8)	C14—C15—H15A	109.6 (10)
C8—C7—H7	108.8 (8)	O2—C15—H15B	109.0 (11)
C9—C8—C7	114.80 (14)	C14—C15—H15B	110.0 (12)
C9—C8—H8A	108.7 (9)	H15A—C15—H15B	107.5 (16)
			
C6—C1—C2—C3	0.8 (3)	C7—C8—C9—C14	104.47 (18)
C1—C2—C3—C4	−0.4 (3)	C14—C9—C10—C11	0.4 (2)
C2—C3—C4—C5	−0.5 (3)	C8—C9—C10—C11	−178.90 (15)
C3—C4—C5—C6	1.1 (3)	C9—C10—C11—C12	0.3 (3)
C2—C1—C6—C5	−0.2 (3)	C10—C11—C12—C13	−0.7 (3)
C2—C1—C6—C7	−176.30 (16)	C11—C12—C13—C14	0.4 (3)
C4—C5—C6—C1	−0.7 (3)	C12—C13—C14—C9	0.3 (3)
C4—C5—C6—C7	175.50 (16)	C12—C13—C14—C15	178.15 (19)
C1—C6—C7—O1	−32.5 (2)	C10—C9—C14—C13	−0.7 (2)
C5—C6—C7—O1	151.38 (15)	C8—C9—C14—C13	178.57 (15)
C1—C6—C7—C8	92.49 (18)	C10—C9—C14—C15	−178.43 (16)
C5—C6—C7—C8	−83.59 (19)	C8—C9—C14—C15	0.8 (2)
O1—C7—C8—C9	−61.90 (19)	C13—C14—C15—O2	118.82 (19)
C6—C7—C8—C9	173.13 (14)	C9—C14—C15—O2	−63.4 (2)
C7—C8—C9—C10	−76.31 (19)		

### Hydrogen-bond geometry (Å, °)

**Table:**

*D*—H···*A*	*D*—H	H···*A*	*D*···*A*	*D*—H···*A*
O2—H2O···O1^i^	0.86 (2)	1.89 (2)	2.745 (2)	170.5 (24)
O1—H1O···O2	0.93 (2)	1.78 (2)	2.706 (2)	173.6 (22)
C15—H15B···Cg(2)^i^	0.95 (2)	2.638 (18)	3.504 (2)	151.7 (14)

Symmetry codes: (i) *x*+1/2, *y*, −*z*+1/2.
